# Impact of Diet and Exercise Interventions on Cognition and Brain Health in Older Adults: A Narrative Review

**DOI:** 10.3390/nu15112495

**Published:** 2023-05-27

**Authors:** Mickeal N. Key, Amanda N. Szabo-Reed

**Affiliations:** 1 KU Alzheimer’s Disease Research Center, Fairway, KS 66205, USA; mkey@kumc.edu; 2 Department of Neurology, University of Kansas Medical Center, Kansas City, KS 66160, USA; 3 Department of Internal Medicine, Division of Physical Activity and Weight Management, University of Kansas Medical Center, Kansas City, KS 66160, USA

**Keywords:** diet, nutrition, exercise, cognition, brain structure, brain function, brain health

## Abstract

The ability to preserve cognitive function and protect brain structure from the effects of the aging process and neurodegenerative disease is the goal of non-pharmacologic, lifestyle interventions focused on brain health. This review examines, in turn, current diet and exercise intervention trends and the collective progress made toward understanding their impact on cognition and brain health. The diets covered in this review include the Mediterranean diet (MeDi), Dietary Approaches to Stop Hypertension (DASH), Mediterranean-DASH Intervention for Neurodegenerative Delay (MIND), ketogenic diet, intermittent fasting, and weight loss management. The exercise approaches covered in this review include endurance, resistance, combined exercise programs, yoga, tai chi, and high-intensity interval training. Although valuable evidence is building concerning how diet and exercise influence cognitive performance and brain structure, many of the open questions in the field are concerned with why we see these effects. Therefore, more strategically designed intervention studies are needed to reveal the likely multiple mechanisms of action in humans.

## 1. Introduction

Cognitive decline is one of the greatest threats to an individual’s independence and quality of life for those 65 years and older [1]. With a large segment of the US population entering this life stage [2], we must use cutting-edge, interdisciplinary research approaches to investigate what factors influence the trajectory of healthy cognitive and brain aging. The literature has identified several critical modifiers of brain and cognitive aging [3]. In addition, the American Heart Association’s “Primary Care Agenda for Brain Health” is based on modifiable risk factors known to affect brain health throughout the lifespan [4]. These modifiable risk factors include aspects of lifestyle such as diet and exercise, both of which have been identified as promising prevention-related interventions [4]. While the focus of prior research on dietary and exercise interventions has traditionally centered on overall health and wellness or cardiovascular health [5,6,7], increasingly, scientists have sought to apply these interventions to improve brain health. Due to the rapidly evolving research in these areas, the focus of this narrative review is to provide an overview of what is known about the impact of diet and exercise on cognition and brain health since the last review of its kind [8,9]. It focuses on their impact as modifiers of cognition and brain aging. A brief review of age-related changes in cognition and brain structure is provided first, followed by a survey of dietary and exercise interventions for impacting cognition and brain structure in older adults.

## 2. Age-Related Changes in Cognition and the Brain

### 2.1. Age-Related Changes in Cognition

Decades of behavioral research have shown that there is significant variance in the cognitive abilities of older adults. Moreover, cognitive aging is typically accompanied by decreased performance within specific cognitive domains and broader cognitive abilities [10,11].

A widely observed age-related change in cognition is reduced processing speed. This manifests from either the inability to access information necessary for problem-solving or simply from an increase in the amount of time needed for task-relevant information processing [12].

Core facets of memory are also affected by the aging process, albeit differently. Take, for example, the two types of conscious, long-term memory, episodic and semantic memory. Episodic memory refers to recalling past experiences (e.g., a recent European trip). In contrast, semantic memory captures general knowledge about the world (e.g., the meaning of “nutrition”). As we reach adulthood, semantic memory performance remains relatively stable compared to episodic memory, which becomes less reliable [13,14,15]. Working memory actively maintains information in short-term memory and enables goal-directed thought and decision-making [15]. However, working memory capacity in older adulthood is known to significantly decline [16]. In addition, working memory enables performance on complex, high-level cognitive tasks [17], and therefore a corresponding reduction in executive functions is also evident in advanced age. Executive functions are higher-level cognitive tasks such as problem-solving and decision-making and measure facets of cognitive control and inhibition [18,19].

In addition to domain-specific changes in cognitive aging, broader facets of cognition are also known to be affected. For example, cognitive abilities that fall under crystallized intelligence (problem-solving in the context of prior knowledge and experience) tend to increase into late adulthood. In contrast, those that fall under fluid intelligence (problem-solving in the context of novel situations) tend to be highly susceptible to aging, peaking in the 20s and 30s and then steadily declining afterward [20,21]. One reason these changes in general and specific facets of cognition occur in late life is due to age-related structural and physiological changes in the brain. The following sections discuss these changes and their connection to the abovementioned cognitive changes.

### 2.2. Age-Related Changes in the Brain

The aging process has a profound effect on the brain [13,22,23,24]. The following sections will discuss age-related changes to brain structure and the underlying changes in physiology that lead to those structural changes.

**Brain Structure.** The aging process differentially impacts brain regions and networks across the cortex. Structural changes in the brain associated with aging include reduced gray matter volume and cortical thickness. The primary regions affected are the prefrontal cortex, hippocampus, medial temporal lobe, and association areas within the parietal lobes [13,22]. In addition, age-related decreases in the integrity of white matter microstructure are evident in anterior cortices and progressing to posterior regions (as measured by decreasing fiber coherence and organization [25].

Gray matter volume is an approximate measure of neuronal and glial cell bodies and can be measured in vivo using structural magnetic resonance imaging (MRI). In older adulthood, gray matter volume and cortical thickness are known to shrink within multiple brain regions, including the prefrontal cortex, association areas of the parietal cortex, and subcortical regions of the medial temporal lobe (i.e., the hippocampus and entorhinal cortex). Research consistently finds that older adults experiencing atrophy in these age-sensitive brain regions also exhibit reduced cognitive performance on tasks of processing speed, memory, and executive function [26,27,28,29]. For example, age-related reduction of hippocampal volume, resulting from a combination of neuronal cell loss and a decrease in neurogenesis, is associated with decreased cognitive performance on memory, spatial learning, and emotional regulation tasks [30]. Additionally, studies reporting a reduction in the prefrontal cortex (PFC) volume also observe declines in performance on tests of executive function [19].

White matter (WM) fibers and tracts are the myelinated axons that physically connect local and distant regions of the brain, thereby facilitating both local and global information processing. In older adulthood, the integrity of the white matter microstructure begins to deteriorate in a non-uniform pattern, with anterior regions changing earlier in the aging process and posterior regions changing much later in the aging process [25]. The integrity of these white matter tracts is often quantified by the existence and severity of white matter hyperintensities (WMH) lesions. WMHs can be identified by structural MRI or with summary measures of fractional anisotropy or diffusivity from magnetic resonance diffusion tensor imaging (DTI).

White matter hyperintensities are understood to be a sign of vascular damage in the brain, representing the lesion of brain tissue. They increase in prevalence with age and are, therefore, more prevalent in older adults than younger adults. Although there is a heritable aspect to developing WMHs, they are also reliably associated with multiple metabolic and cardiovascular risk factors. In addition, WMHs are related to structural and functional brain changes such as reduced frontal lobe metabolism and worsening executive function scores [31].

Diffusion tensor imaging (DTI) is a neuroimaging technique that examines the structural integrity of white matter tracts throughout the brain. Tract integrity can be measured by assessing the physical properties of fiber organization and water molecule movement along primary, secondary, and tertiary diffusion axes [32]. Fractional anisotropy (FA) measures fiber coherence and is calculated as a ratio of diffusion in the primary orientation compared to other orientations. A region with a high preference for a particular orientation will have a high FA value, indicating highly organized fibers, a sign of intact white matter tracts [32]. FA decreases in older adulthood and provides evidence for decreased fiber integrity in late life [33]. Axial diffusivity measures diffusion along the primary orientation, radial diffusivity measures diffusion along the secondary and tertiary orientations, and mean diffusivity is the mean diffusion along all three orientations. Diffusivity increases with age, indicating decreased fiber organization and therefore decreased tract integrity [25]. DTI studies have shown that increases in diffusivity (a measure of disordered fiber orientation) and reduced fractional anisotropy (reduced microstructure integrity), mostly in frontal regions and were linked to declines in executive function [34] and fluid intelligence [35].

Additionally, the neurobiology of aging literature demonstrates that other neuronal circuits vulnerable to the effects of aging are located in the hippocampus and neocortex. The neurons in these circuits tend to be pyramidal and have connections to the prefrontal, temporal, and parietal areas [36]. Dendritic spines of pyramidal neurons have also been found to be especially affected by age [37]. The vulnerability seems to be the loss of synapses and synaptic plasticity in these regions. Synaptic plasticity is the bedrock of learning and memory, making it especially important for higher-level cognitive functions in humans.

The structural brain changes described here support the literature on cognitive aging. As mentioned above, gray matter atrophy is associated with decreased memory performance, white matter hyperintensities have been associated with reduced executive function and poor white matter integrity is associated with disrupted network connectivity, leading to slower processing speed and reduced executive control consistently observed in older adults [25,38]. It is important to note that these age-related structural changes do not occur in isolation but are often exacerbated by genetic risk factors, environmental risk factors, and disease processes such as cardiovascular disease [39].

**Brain Physiology.** Along with the regional age-related structural gray and white matter changes in the brain, there are also age-related physiological changes with a more global effect across the brain. The primary physiological changes addressed in the neurobiology of aging literature include energy metabolism, calcium homeostasis, immune function, and growth factors. These global brain changes not only impact the trajectory of brain volume and white matter integrity throughout the aging process but also hold deleterious consequences for cognitive performance [40]. Each of these neurobiological mechanisms is described in turn below.

One important age-related change in brain physiology is in energy metabolism. With normal aging, mitochondria become dysfunctional and glucose metabolism is altered. Iron plays a lead role in energy metabolism due to its involvement in the electron transport chain, which produces ATP in mitochondria. Complex-bound iron is safe in the mitochondria, but the aging process makes this binding process inefficient, leaving unbound iron in the mitochondria. This leads to mitochondrial dysfunction (reduced energy output) that also produces damaging reactive oxygen species/free radicals that damage neural cell membranes. Reduced energy sources also inhibit cellular repair mechanisms and eventually cause loss of neuropil and myelin [40], leading to atrophy in the periphery of the brain vasculature and regions such as the dorsolateral prefrontal and inferior parietal cortices [24]. These structural changes result in reduced information processing capacity of brain networks, impairing cognitive processes, with the most complex, least automated processes being more susceptible to this system-wide noise [40].

Calcium homeostasis is another physiological process impacted by aging. Calcium’s movement through plasma membranes, intercellular concentrations, and its use as metabolic buffers and sensors are all crucial to neuronal function. Therefore, it is tightly regulated by neurons. As the aging process can affect these mechanisms, the consequences are widespread, disrupting neurotransmitter release, neuronal excitability, synaptic plasticity, gene expression, programmed cell death, and other metabolic processes in the brain [41]. In addition, these disruptions have implications for cognitive functions such as learning and memory due to their dependence on molecular mechanisms activated by calcium signalling.

A crucial explanatory framework for understanding the effects of inflammation on the aging brain is inflamm-aging, described in the literature as the progressive increase in systemic inflammation as humans age [42]. As we age, pro-inflammatory proteins increase while anti-inflammatory proteins decrease [43]. In addition, the aging process results in higher levels of oxidative stress due to lipid peroxidation [44,45]. Gone unchecked, inflammation and free radicals can damage neurons and synapses, which profoundly affect cognitive function [45]. In a longitudinal study of over 1800 healthy older adults, ten inflammatory proteins were correlated with processing speed, attention, and memory measures over a 6-year follow-up period [46]. Converging evidence from a second longitudinal study of approximately 1000 older adults found that individuals with low LDL cholesterol and high levels of inflammation had lower scores for tests of general cognition and memory [47].

Lastly, older adulthood is associated with changes in growth factors and neurotropic factors. Brain-derived neurotrophic factor (BDNF) is an essential protein in the brain that is required for neuronal health, brain development, learning and memory. The aging process can lead to dysregulation of BDNF signaling, causing atrophy and declines in cognitive performance [48]. BDNF also has a neuroprotective role, protecting the brain against oxidative stress [49]. Healthy lifestyle factors such as diet and exercise have also been associated with increases in BDNF to facilitate their beneficial effects on cognitive aging [49,50].

**Summary.** Collectively, age-related changes in cognition, brain structure, and brain physiology operate as potential targets for lifestyle interventions, especially those focused on a healthy diet and exercise, aimed at improving age-related cognitive outcomes and possibly aiding in the prevention or delay of cognitive decline related to Alzheimer’s and related dementias. The upcoming section describes how nutrition impacts brain health, supporting cognitive function in older adults.

## 3. Effects of Diet on Cognition and Brain Health in Aging

Evidence from the scientific literature is clear that there are key lifestyle factors that have an essential impact on brain health. For example, the American Heart Association and the American Academy of Neurology have stated that Life’s Simple 7^®^ Modifiable risk factors across the life course, first established for cardiovascular health, are just as appropriate for brain health [4]. Since food is required to sustain life, a healthy diet is a prime modifiable behavior on which to focus. In addition, a healthy diet can also help an individual with other lifestyle factors included in Life’s Simple 7^®,^ including weight control, lipid control, blood pressure control, and reduction of diabetes risk [4]. The question then becomes, what is a healthy diet? To answer this, it is essential first to understand the important role nutrients play in neuronal function, neurometabolic processes, and, ultimately, cognitive function. Considerable evidence in nutritional epidemiology demonstrates that essential amino acids, fatty acids, vitamins, and minerals are critical for the proper function of the body [51,52]. Amino acids in the diet come primarily from consuming meat, while many of the vitamins and minerals come from various fruits, vegetables, beans, and nuts [51,52]. The brain relies upon these nutrients to support a variety of functions related to neuronal health and survival, nerve impulse, synthesis of neurotransmitters, lipid membrane asymmetry and integrity, synaptic plasticity, and a wide array of metabolic functions related to energy and homocysteine production [44,53,54]. These processes are critical to brain health and intimately tied to cognitive function.

This accumulated evidence has resulted in numerous dietary interventions that use supplementation with individual nutrients or select groups of nutrients to examine their effect on reversing or stopping cognitive decline once clinical signs appear. Unfortunately, these studies are either inconclusive or show no therapeutic effect. Examples include studies examining: Vitamin E [55]; Vitamins & Minerals [56]; Folic Acid with/without B12 [57]; Vitamin D [58]. However, new evidence from a randomized clinical trial provides evidence for the role of a multivitamin/mineral supplement in supporting global cognitive performance in older adults, especially those with a history of cardiovascular disease [59].

### 3.1. MeDi, DASH, MIND Diets

A growing body of evidence suggests that comprehensive, whole diets are most effective in delaying or reducing the incidence of cognitive decline and dementia [60]. The prevailing thought is that studying whole diets or components of diets comes from an understanding that individuals eat various foods across multiple food groups, and the nutrients from those foods have synergistic effects above and beyond the effects associated with individual nutrients or nutrient groups. As an example, the absorption of vitamins in a dark leafy green salad is improved when eaten with olive oil and vinegar instead of a fat-free ranch dressing. Here, healthy fats in olive oil are a carrier for the vitamins and minerals in the leafy greens [54]. This example illustrates the need for a more ecologically valid approach, where nutrient status is based on more holistic dietary patterns.

Several recent reviews identify multiple observational studies (both cross-sectional and longitudinal) and intervention trials that provide consistent and converging evidence for the positive impact of the Mediterranean diet (MeDi), the Dietary Approaches to Stop Hypertension (DASH) diet, and the Mediterranean-DASH Intervention for Neurodegenerative Delay” (MIND) diet on brain health and cognition [60,61,62]. By far, these dietary patterns are the most referenced in the literature. Although these diets were developed for different purposes, their components overlap significantly. Here we describe these three diets and summarize the literature associated with their impact on cognition and brain health.

#### 3.1.1. Mediterranean Diet (MeDi)

The MeDi focuses on the traditional dietary patterns of countries near the Mediterranean Sea and emphasizes fruits, vegetables, nuts & seeds, and low-fat dairy [63,64]. The diet is generally highly regarded for its contribution to cardiovascular health. In addition, the MeDi includes recommendations for physical activity and social engagement.

#### 3.1.2. Dietary Approaches to Stop Hypertension (DASH) Diet

The DASH diet was developed to help individuals with hypertension reduce their sodium intake to improve blood pressure and is focused on increasing fruit, vegetables, whole grains, and nuts. In addition, the diet suggests poultry and fish for meat intake and dairy should be low-fat. The diet also encourages salt restriction and limited consumption of red meat, sweets, and added sugars in drinks [65]. The literature shows that the DASH diet is also an effective approach to reducing the risk of cardiovascular disease [66].

#### 3.1.3. “Mediterranean-DASH Intervention for Neurodegenerative Delay” (MIND) Diet

Building on the established research of the MeDi and DASH diets, Morris and colleagues developed the “Mediterranean-DASH Intervention for Neurodegenerative Delay” (MIND) diet, a combination of the Mediterranean diet and the Dietary Approaches to Stop Hypertension (DASH) diet [67]. The MIND diet measures 15 dietary components, ten healthy components, and five unhealthy components, each scored according to consumption frequency, with higher scores obtained by consuming more healthy and less unhealthy foods. The healthy components of the MIND diet emphasize the consumption of vegetables, especially leafy green vegetables. It is also suggested to eat berries (instead of fruit), nuts, beans, legumes, whole grains, lean meat, and wine. In addition, the MIND diet emphasizes using olive oil as the primary cooking fat.

#### 3.1.4. Dietary Components shared by MeDi, DASH, and MIND

**Vegetables.** Dark leafy greens and other vegetables are rich in vitamins A, C, E and K, folate, carotenoids, fiber, iron, magnesium, potassium, and calcium. Evidence suggests that these nutrients promote vascular health, and the antioxidants serve a neuroprotective role [5,68]. This is primarily why they are investigated for their role in cognition [69]. For example, a study by Morris and colleagues found that a reduction in cognitive decline was associated with consuming at least one serving per day of green leafy vegetables [68].

**Fruits and Berries.** The flavonoids in berries have been associated with reduced cognitive decline [70]. This is attributed to the anti-inflammatory and antioxidant properties of flavonoids, along with their involvement in neuronal signaling and accumulation in brain regions that support learning and memory [70,71].

**Nuts and Olive Oil.** Fatty acids such as omega-3 polyunsaturated fatty acids (PUFAs) and polyphenols are found in abundance in a variety of nuts and olive oil [70,72]. Dietary fatty acids are anti-inflammatory agents and support a healthy vascular system [73]. Dietary polyphenols can also improve cognitive function by supporting neuronal signaling and acting as antioxidants and anti-inflammatory agents [74]. Together these bioactive nutrients support cognitive function by protecting brain structure and metabolism.

**Whole Grains and Beans**. Whole grains and beans are an essential source of complex carbohydrates, fiber, and vitamin E. While they have not been tied to brain health directly or indirectly, they play a supporting role in maintaining a healthy weight, gut, and cardiovascular system [75,76,77]. At a systemic level, this helps reduce key risk factors often associated with dementia, such as diabetes, cardiovascular disease, and obesity [75,76,77].

**Dairy**. Recent research on dairy consumption and brain health suggests that low-fat and fermented dairy products may offer beneficial cognitive effects and reduce the risk of dementia later in life [78]. Low-fat dairy contains protein that supports glucose regulation and insulin release, bioactive peptides that support metabolic and immune processes, metabolism-supporting vitamins and minerals like vitamin B12 and calcium, and probiotics [79]. In a recent randomized control trial, Choi and colleagues examined the effect of milk consumption on the concentration of glutathione, a brain antioxidant, in a cohort of older adults with low dairy consumption rates. Compared to the control group, who maintained low dairy consumption, the intervention group consumed 3 cups of 1% milk daily (as suggested by the Dietary Guidelines for Americans). It significantly increased glutathione at the end of the 3-month trial [80]. Additionally, fermented dairy products contain bioactive agents such as oleamide and dehydroergosterol, which facilitate the reduction of microglial activation and neurotoxicity [81].

**Lean Meat and Fish.** Macro-nutrients such as the proteins and amino acids found in lean meat and fish have long been studied for their function in cognitive aging due to their significant contribution to energy metabolism and their function as precursors for neurotransmitters (e.g., serotonin, dopamine, and norepinephrine) required for mood, motivation and attention [82,83]. Dietary neurotransmitters such as acetylcholine and glutamate are found in seafood and are essential for learning and memory [82,83]. Fish and seafood are also excellent sources of mono- and poly-unsaturated fatty acids [72,73]. Dietary intervention studies have shown that Omega-3 PUFAs increase brain-derived neurotropic factor (BDNF), which is known to improve synaptic function and support cognitive mechanisms for memory processing [74,84,85]. Lean meat and fish are also excellent sources of vitamins B12 and D, essential for the proper functioning of the nervous system, as evidenced by nervous system dysfunction when a deficiency in either of these vitamins is present [5].

**Red Meat, Fried Foods, Fast-Food, Pastries, and Sweets.** The MeDi, DASH, and MIND diets all suggest the restricted intake of red meat, fried foods, fast foods, pastries and sweets. Morris calls these “brainless foods” in her 2017 book, where she stresses the importance of reducing the consumption of red meat, full-fat dairy, fried food, fast food, pastries, and sweets [86]. Saturated fats are one of the main reasons for limiting the consumption of red meat and fried foods. Saturated fats are connected to an increased risk of cardiovascular disease and developing dementia in late life [87]. In mice, saturated fat consumption is associated with reduced blood-brain barrier integrity and hyperactive microglia [88]. In addition, red meat and fast food are often cooked using high heat and therefore leads to increased consumption of advanced glycation end-products (AGEs), which increase inflammation and promote the proliferation of amyloid beta plaques and neurofibrillary tangles [72,89,90,91,92].

Furthermore, fried foods and fast food typically contain added trans fats and sodium, magnifying their negative impact on the cardiovascular system [93,94,95]. Pastries and sweets are simple carbohydrates that are a hallmark of the Western diet, with overconsumption linked to insulin sensitivity and obesity [96]. Both chronic conditions are risk factors for dementia in late life [97].

#### 3.1.5. Effects of the MeDi, DASH, and MIND Diets on Hallmarks of the Aging Brain

When considering the systemic effects of aging on the brain and how individual nutrients support brain function (see review above), it is reasonable to expect that the MedDi, DASH, and MIND diets may exert a systemic effect on the brain, associated with the hallmarks of the aging [98]. These systemic effects include (1) cell membrane and vascular integrity, (2) inflammation, resolution, and oxidation, and (3) lipid and energy metabolism. Each of these factors is reviewed in turn below.

**Cell Membrane and Vascular Integrity.** The Mediterranean diet [99,100] and the DASH diet [101,102] have both been found to preserve the structural integrity of cellular membranes and vasculature by supporting cardiovascular and metabolic health in observational studies. Although the MIND diet has not yet been investigated in this regard, much like the diets it is derived from, it likely supports healthy cholesterol and triglycerides, low blood pressure, and reduced coronary artery disease due to its focus on consuming foods high in whole grains and omega-3 fatty acids [103,104].

**Inflammation, Resolution, and Oxidation**. The aging process is accompanied by an increase in pro-inflammatory proteins and a decrease in anti-inflammatory proteins [43]. Inflammation and free radicals can damage neurons and synapses necessary for information processing across the brain. This loss of neuronal function results in a profound effect on cognitive function [45].

Vitamins (like vitamins B_1_, B_6_, B_12_, D, and folate) and minerals (such as calcium, magnesium, zinc, and selenium) that act as anti-inflammatory and antioxidant agents protect and preserve brain structures from damage due to the generation of reactive oxidative species by mitochondria and lipid peroxidation, and neural insults due to chronic inflammatory molecules [54,69].

At the same time, the aging process is also associated with a decreased ability to resolve inflammation, inhibiting the repair and restoration of damaged tissue and clearance of cellular waste and debris [105]. Resolution of inflammation, which includes the suppression of pro-inflammatory molecules, decreased permeability of the vasculature, and increased macrophage clearance of debris, depends on specialized pro-resolving mediators (SPMs) derived from omega-3 fatty acids. Preclinical studies have identified omega-3 fatty acid supplementation as a promising intervention. But evidence from randomized clinical trials has been inconclusive, with supplementation only helping a subset of participants [105,106].

Many cross-sectional studies appeal to nutrition’s anti-inflammatory/antioxidant benefits to explain why a particular diet or group of nutrients or diet is associated with better cognition [67,68,107,108,109]. A recent review highlighted studies that have incorporated biomarkers of inflammation as an outcome, finding that cross-sectional assessments consistently supported the association between higher diet scores and lower inflammatory markers [110]. Although it has yet to be investigated, the MIND diet’s emphasis on foods high in anti-inflammatory compounds (berries and dark leafy greens) and omega-3 fatty acids (salmon and olive oil), it is reasonable that high adherence would be associated with reduced inflammation and oxidation [84].

**Lipid and Energy Metabolism**. Age-related changes to neurotransmitter function, energy production, and nutrient utilization can also drastically change the brain, reducing the efficiency of information transmission across cortical networks [111,112,113]. Diets that provide significant omega-3 fatty acids and polyphenols have been shown to increase levels of BDNF, an essential molecule for brain function due to its role in normal neural function, moderation of energy homeostasis and metabolism, and its neuroprotective properties [84]. BDNF preferentially accumulates in the hippocampus and cerebral cortex, which supports learning and memory by promoting synaptic plasticity [74]. Additionally, BDNF can protect against amyloid beta (Aβ) toxic effects and tau hyperphosphorylation in Alzheimer’s disease, despite concurrent Aβ-mediated suppression of BDNF mRNA [114]. Although it is currently unknown how the DASH and MIND Diet might influence BDNF levels, a study examining the MeDi found that the diet was not significantly associated with BDNF levels in the full sample but was significant in a subsample of depressed individuals [115].

In summary, likely, multiple components of the MeDi, DASH, and MIND diets that lead to the increased consumption of vitamins, minerals, polyphenols, and omega-3 fatty acids may have individual and additive effects that target systemic factors associated with brain aging.

#### 3.1.6. Effects of the MeDi, DASH, and MIND Diets on Cognition in Aging

**Global Cognition and Neurodegenerative Disorders.** A review by van den Brink and colleagues found that higher adherence to the MeDi, DASH, and MIND diets was associated with better cognitive scores [60]. Further, recent reviews show adherence to the MeDi diet was associated with decreased risk of developing mild cognitive impairment (MCI), Alzheimer’s Disease (AD), dementia, and conversion from MCI to AD [99,116]. Although not as well studied as the MeDi, there is also evidence to suggest that MIND diet adherence is associated with a reduced risk of AD and MCI. [117]. Morris and colleagues examined data from a cohort of 960 older adults. They found that even with a median MIND score of 9.5 out of 15, individuals in the highest tertile of MIND scores had slower rates of cognitive decline over ten years than those in the lower tertiles. The authors found that those with the highest MIND diet scores had a lower prevalence of cardiovascular conditions, higher physical activity, and higher education levels. Even in models including these covariates, the relationship between MIND diet scores and slower cognitive decline remained [67]. Additionally, the authors examined the same cohort and found that higher MIND diet scores were associated with a decreased risk of developing AD over ten years. The estimated effect of the diet was a 53% reduction of risk for individuals in the highest tertile (mean MIND diet score of 9.6 out of 15) and 35% for the middle tertile (mean MIND diet score of 7.5 out of 15) [117]. Although there are no intervention studies that manipulate MIND diet adherence to examine cognitive performance in older adults with or without cognitive challenges, there is a study currently in progress to address such questions [118].

**Domain Specific Cognitive Performance.** While the MeDi, DASH, and MIND diets have been associated with benefits on global cognition, prior research has also investigated their association with domain-specific cognitive processes in aging. Research supports that both the MeDi & MIND diets have a positive effect on working memory and verbal fluency [99,119,120,121]. The two diets also have a separate effect on other domain-specific cognitive functions. The MeDi diet has been positively associated with attention [122,123] and long-term memory [124]. MIND diet adherence has been positively associated with performance on measures of visuospatial ability [67,125], perceptual speed [67,121], and executive function [67,123,125,126].

**Summary.** A large body of evidence suggests that the MeDi and MIND diets are associated with both general and domain-specific facets of cognitive ability. Overall, the effects of these diets on global cognition are attributed to a specific focus on the intake of berries, leafy green vegetables, whole grains, nuts, and olive oil. As described previously, the flavonoids in berries, extensive vitamins and minerals in leafy greens, and omega-3 fatty acids in fatty fish and olive oil have well-established anti-inflammatory, antioxidant, and other neuroprotective properties [5,68,70,71]. On the other hand, the more domain-specific effects of the MeDi and MIND diet may be attributed to the fact that certain brain regions are more vulnerable to the effects of aging. Therefore the effects of flavonoids, vitamins, minerals, and omega-3 fatty acids can exert a local effect in addition to their systemic effect [60,67,127].

### 3.2. Effect of Ketogenic Diet and Intermittent Fasting on Cognition & Brain Function

Two other diets examined in the literature in relation to cognition and brain function include the ketogenic diet (KD) and intermittent fasting (IF). The ketogenic diet pushes the body’s metabolism into a state of ketosis, where the body switches to using ketones as a primary energy source instead of glucose. This can be accomplished by decreasing carbohydrate intake, increasing fat intake, or taking a ketogenic supplement [128]. In a recent review of 10 randomized control trials, ketogenic diet adherence was associated with improved general cognition and episodic memory in patients with MCI and AD [129]. Intermittent fasting alternates between a fasting and non-fasting state, with the fasting state lasting at least 12 h [130]. Recent reviews have also connected intermittent fasting with improved cognition for healthy adults [131] and those with dementia [132]. The mechanism for both diets is believed to be improvements at the cellular level, with the KD associated with ketosis and IF related to caloric restriction. In both cases, the result is neurotransmitter regulation, synaptic maintenance, and oxidation-reduction, although much of this work is in animals [133,134]. Although not new diets, both the ketogenic diet and intermittent fasting are new approaches in the field of nutritional interventions to improving cognition and brain health in humans, so more intervention work will be required to understand the advantages and disadvantages of adhering to these diets and who may benefit the most from adopting them.

### 3.3. Effect of Weight Management Diets on Cognition & Brain Function

Several studies have noted that improved cognitive functioning is associated with intentional weight loss. Brinkworth et al. [135] reported enhanced working memory and processing speed in a sample of overweight and obese individuals who lost weight on either a low-carbohydrate/high-fat or high-carbohydrate/low-fat diet over 1 yr. Smith et al. [136] showed that participants on the DASH diet combined with a behavioral weight management program exhibited more significant improvements in executive function, memory, learning, and psychomotor speed. In addition, DASH diet alone participants showed better psychomotor speed compared to when with usual diet control. Neurocognitive improvements appeared to be mediated by increased cardiovascular fitness and weight loss. Siervo et al. [137] reported weight loss in obese (BMI 30–50) individuals was associated with improved cognitive performance as assessed by the Trail Making Test. Bariatric surgery-induced weight loss has also been show to enhance attention, executive function and memory [138]. Gowey and colleagues [139] found that individuals with obesity who achieved clinically significant weight loss via a behavioral intervention have average to above-average executive function. Similarly, individuals who maintained their weight loss for at least one year, compared to those who regained, performed better on decision-making tests. Recently, Szabo-Reed et al. found that stronger baseline attention was associated with completing a 3-mo. weight loss intervention, executive control, and working memory were related to the weight loss achieved [140].

In addition to diet alone, adding exercise has also been shown to positively affect cognition. Peven et al. [141] enrolled adults with overweight and obesity into a 12-month behavioral weight loss intervention. Participants were assigned to either an energy-restricted diet alone, an energy-restricted diet plus 150 min of moderate-intensity exercise per week or an energy-restricted diet plus 250 min of exercise per week. Following the intervention, weight significantly decreased in all groups. The authors found a significant multivariate effect of group on cognitive changes and a Group × Time interaction only on Iowa Gambling Task (IGT) reward sensitivity, such that the high exercise group improved their performance relative to the other two intervention groups. There was also a main effect of Time, independent of the intervention group, on the IGT net payoff score. Changes in weight were not associated with other changes in cognitive performance. Overall, the authors concluded that engaging in a high amount of exercise improved reward sensitivity above and beyond weight loss alone. This suggests an additional benefit to adding exercise into behavioral weight loss regimens on executive functioning, even without additional weight loss benefits.

### 3.4. Limitations of Available Research

The primary limitation of available research is the lack of evidence via intervention trials. Most of what is known in the literature concerning the relationship between diet, cognition, and brain health is due to observational studies, the majority of which are cross-sectional, with a few longitudinal. Also, most studies focus on cognitive performance as a primary outcome, whereas few have explored the neural mechanisms underlying improvements in cognition in an intervention.

### 3.5. Dietary Intervention Summary

Dietary patterns are an essential factor in cognitive and brain health for aging individuals. Common targets of all diets addressed include inflammation, oxidation, glucose metabolism, insulin sensitivity, and adiposity, all factors associated with aging and considered risk factors for Alzheimer’s disease and related dementias. Table 1 provides a brief overview of each dietary intervention approach described above.

## 4. Effects of Exercise on Cognition and Brain Health in Aging

In addition to diet, physical activity and exercise have a biologically plausible and temporal relationship with a multitude of diseases, including coronary heart disease [142], atherosclerosis [143], stroke [144], type 2 diabetes [145], some cancers [146], and all-cause mortality [147,148]. Physical activity is any bodily movement produced by skeletal muscles that requires energy expenditure. Exercise, on the other hand, is a subset of physical activity that is planned, structured and repetitive and has the improvement or maintenance of physical fitness [149]. Regular endurance and resistance exercise training decreases age-related morbidity and mortality, improves risk factors for chronic disease, and helps maintain independent functioning [147,148,150].

### 4.1. Brain Mechanisms Associated with Exercise

Animal research suggests that exercise positively impacts brain health [151,152,153,154,155,156,157,158,159,160,161,162,163,164,165,166]. Specifically, exercise stimulates neurogenesis [151], as evidenced by increased counts of new neurons in adult animals on an exercise regimen. Exercise is also associated with enhanced neuronal survival [152], resistance to brain injury [153,154], and increased synaptic development and plasticity [155]. Exercise promotes vascularization in the brain [156,157], is associated with increased learning [151,158], mobilizes gene expression profiles predicted to benefit brain plasticity [159], and maintains cognitive function [160]. Exercise in cognitively normal older adults is associated with evidence of lower cerebral amyloid deposition (as assessed by both brain PET PIB imaging and CSF Aβ) [162,165,167]. Exercise may modulate vascular risk factors for dementia (atherosclerosis [143], heart disease [142], stroke [144], diabetes [168,169,170,171,172,173]). Studies have specifically shown that exercise decreases systemic inflammatory markers [174] and increases levels of endogenously-produced, neuroprotective proteins such as brain-derived neurotrophic factor (BDNF) that support neuronal growth and survival [175,176]. Exercise also positively affects energy balance and glucose metabolism via actions on AMP kinase and insulin signaling, processes that have been suggested to increase Aβ trafficking and clearance [177,178,179].

### 4.2. Endurance Exercise and Cognition/Brain Structure

Endurance exercise consists of prolonged physical exertion with energy requirements supplied primarily by endurance metabolism. Public health recommendations from the World Health Organization (WHO), Centers for Disease Control (CDC), American College of Sports Medicine (ACSM), as well as others, recommend that older adults do at least 150 min of moderate-intensity endurance exercise per week (46–63% of maximal oxygen consumption capacity [VO_2_max]) as part of a regular exercise regimen to maintain health and fitness [150,180,181]. Endurance exercise generally consists of walking, jogging, running, swimming, and cycling, with walking being the most practiced form of endurance exercise among older adults [182]. Endurance exercise regimens produce beneficial physiologic adaptations in older adults, including increases in cardiorespiratory fitness, metabolic adaptations with benefits to glycemic control and lipids, and reduced body fat [150].

Most studies of the effect of exercise on brain health focus on endurance exercise or physical activity, reflecting predominantly endurance-type activities. Observational studies have demonstrated that self-reported physical activity is positively associated with cognitive differences at baseline or may drive longitudinal gains or slower decline over time [183,184,185,186,187,188]. Additionally, MRI studies suggest that exercise, and associated endurance fitness levels, may attenuate age- and AD-related brain changes. Higher endurance fitness levels are associated with less age-related brain volume decline [189,190,191].

Randomized controlled trials have examined the role of endurance exercise on cognition. Though the results are mixed, the overall evidence suggests that endurance exercise in healthy, older adults may have a beneficial impact on cognitive performance [192,193,194,195,196,197], promotes brain plasticity [193,198], and attenuates hippocampal atrophy while improving visual attention and memory [193]. A meta-analysis [199] examined 18 endurance intervention studies of varying quality and found a moderate effect for combined exercise programs across all cognitive outcome measures (effect size = 0.6). Increasing age did not appear to attenuate these benefits, with evidence that individuals aged 71 to 80 had perhaps greater benefits than younger age groups.

### 4.3. Resistance Training and Cognition

Resistance training is an important component of a complete exercise program for older adults [200]. It uses muscular contraction against resistance to mitigate the effects of aging on neuromuscular function and functional capacity [201,202,203,204,205]. It can also improve muscle strength, mass, and output [206]. Older adults retain the ability to benefit from resistance exercise to a similar extent as younger adults [150]. In addition to endurance exercise, public health recommendations suggest that older adults perform resistance training at least twice weekly to maintain function, health, and fitness [207]. Physiologic benefits include increased muscle mass and power and bone mass and strength [208]. These benefits of resistance exercise are not consistently observed with endurance exercise and are critical for maintaining function and combating age-related sarcopenia [209,210]. Bioenergetic adaptations from resistance training include increasing high-energy phosphate (ATP and creatine phosphate) availability and increasing mitochondrial density and oxidative capacity [150].

There are fewer large, well-designed, randomized controlled trials assessing resistance training on brain health outcomes, although the available literature has proved promising [200]. Randomized clinical trials have examined the effects of resistance training on cognitive function and have found that participation results in improvements in executive function [211], memory [212], verbal fluency [212], and global cognition [212,213,214]. However, results have been inconsistent in showing that resistance training can prevent cognitive decline and AD [215,216]. In a study of 62 older adults randomized to resistance training or a control group, resistance training (both high and low-intensity groups) was associated with improved working memory [217]. In another study of 155 older women [218], one year of resistance training was associated with the benefit of selective attention and conflict resolution performance compared to those randomized to the control group. Paradoxically, resistance training was associated with a 0.3–0.4% decline in whole brain volume compared to controls, though this effect has yet to be replicated. A recent systematic review showed that resistance training positively affected older adults’ executive cognitive ability and global cognitive function. It also had a weak but positive impact on memory. There was no significant improvement in attention. The authors also concluded that tri-weekly resistance training has a better effect on general cognitive ability than biweekly [219].

### 4.4. Combined Exercise and Cognition

Despite the widespread recommendation for combined exercise, no studies have directly compared the effects of aerobic vs. resistance or combined training on cognition. However, studies have assessed the differential impact of these exercise modalities on body weight and composition [220,221,222], insulin resistance [222,223,224,225,226], inflammation [227], and functional limitations [223,224,225]. The results of these studies suggest that combining aerobic and resistance training is optimal for effects on insulin resistance [223,225] and physical function [225] but does not offer advantages for altering adiposity [228].

Resistance and endurance training elicit physiologic adaptations to cardiovascular, muscular, bioenergetic, and neuroendocrine systems [217,229,230]. Resistance training relies preferentially on anaerobic metabolism during the short but intense training bouts. This improves muscle strength and quality while increasing high energy phosphate (ATP and creatine phosphate) availability, mitochondrial density, and oxidative capacity [150], effects that are generally not observed with aerobic exercise. In contrast, aerobic exercise training increases the capacity of muscle to generate energy through increased myoglobin content in muscle and increased efficiency of oxygen extraction and carbohydrate oxidation. Despite some concern that combined aerobic and resistance training will result in an “interference effect” where the development of strength during the same period might influence the development of aerobic capacity and vice versa, several studies have found no evidence of this possible effect [227,230].

The field has not directly assessed whether public health recommendations provide independent or combined effects on cognition in older adults. Conclusions from prior work are limited by design. Specifically, there is limited literature comparing resistance or combined exercise to a non-exercise control [218,231,232,233,234,235,236,237,238]. There is also high variability in endurance exercise types: walking, circuit training, running [239], swimming/aqua endurances [239], etc. [194,215,240]. There is also variability in resistance training parameters, including modality, weekly sessions, and progression [218,232,234,235,241,242]. Finally, there is an ongoing trial to test the independent and combined effects of resistance and endurance training on brain health and physiology in old adults [243].

### 4.5. Other Forms of Exercise

**Yoga**. Yoga is a popular complementary health approach and form of physical activity practiced by adults and older adults. Yoga combines physical postures, rhythmic breathing, and meditative practice to offer those who do it a unique holistic mind-body experience. A recent systematic review and meta-analysis evaluated the effect of yoga-related mind-body therapies on cognitive function in older adults. For example, Bhattacharyya, Andel and Small [244] found 12 studies and 11 randomized controlled trials. The studies involved various yoga practices with a common focus on meditative postural exercises. They revealed significant beneficial effects on memory (Cohen’s *d* = 0.38), executive function (Cohen’s *d* = 0.40), and attention and processing speed (Cohen’s *d* = 0.33).

Similarly, Gothe et al. [245] reviewed 11 studies examining the effects of yoga practice on brain structures, function and cerebral blood flow. The studies demonstrate a positive effect of yoga practice on the structure and/or function of the hippocampus, amygdala, prefrontal cortex, cingulate cortex, and brain networks, including the default mode network. However, there is variability in the neuroimaging findings that partially reflects different yoga styles and approaches and sample size limitations [246]. Overall, the existing body of research offers early evidence that behavioral interventions like yoga may hold promise to mitigate age-related and neurodegenerative declines, as many of the regions identified are known to demonstrate significant age-related atrophy.

**Tai Chi**. Tai Chi is another popular complementary health approach and form of physical activity practiced by adults and older adults. Tai Chi is a traditional Chinese martial art that includes a series of slow, gentle movements, physical postures, a meditative state of mind and controlled breathing. Research surrounding this mind-body exercise suggests it may impact older adults’ cognition and brain function. For example, Liu et al. [247] recently completed a systematic review and meta-analysis to evaluate the impact of Tai Chi on cognitive function. The authors found Thirty-three randomized controlled trials and that tai chi could progress global cognition when assessed in middle-aged and elderly patients suffering from cognitive and executive function impairment. Similarly, a recent literature review to evaluate the effect of tai chi practice on brain structure and neurobehavior changes found the increased volume of cortical grey matter, improved neural activity and homogeneity, and increased neural connectivity in different brain regions, including the frontal, temporal, and occipital lobes, cerebellum, and thalamus. Furthermore, the longer one practices tai chi, these brain regions are altered [248].

**High-Intensity Interval Training (HIIT)**. High-intensity interval training (HIIT) has emerged as a time-efficient strategy to improve health-related fitness compared to traditional training methods. HIIT is an interval exercise that incorporates several rounds of alternating exercises at a high intensity (i.e., 80% of heart rate max) followed by a short period of lower-intensity movements (i.e., recovery). Leahy et al. [249] recently conducted a review to explore the impact of HIIT training on cognitive function in children and adolescents. A total of 22 studies were included in the review. Acute studies showed small to moderate effects for executive function (standardized mean difference [SMD], 0.50, 95% confidence interval [CI], 0.03–0.98; *p* = 0.038) and affect (SMD, 0.33; 95% CI, 0.05–0.62; *p* = 0.020), respectively. Chronic studies also showed a small significant effect on executive function (SMD, 0.31; 95% CI, 0.15–0.76, *p* < 0.001), well-being (SMD, 0.22; 95% CI, 0.02–0.41; *p* = 0.029), and ill-being (SMD, −0.35; 95% CI, −0.68 to −0.03; *p* = 0.035). The review provides preliminary evidence suggesting that participation in HIIT can improve cognitive function and mental health in children and adolescents. Recent evidence also supports the contention that HIIT elicits higher fat oxidation in skeletal muscle than other forms of exercise and is an excellent stimulus to increase maximal oxygen uptake (VO_2_ max). HIIT also seems to be an excellent stimulus to enhance BDNF (a protein synthesized in neurons that participates in cognitive processes as measured at the hippocampus) [250]. In addition, HIIT should be included in stroke rehabilitation for its beneficial effects on neuroplasticity processes [251]. HIIT has also enhanced cognitive flexibility in older adults [252]. The findings in older mice suggest HIIT can improve physical function and reduce frailty, decreasing the risk of disability and loss of independence with age [253,254]. However, more research on HIIT is needed before strong conclusions can be drawn.

### 4.6. Limitations of Available Research

Although the literature supporting the influence of exercise on cognition and brain health appears robust, there are several limitations of this review and the literature itself that should be mentioned. First, this is not a systematic review; all the available literature is not represented. Current public health recommendations state that older adults do at least 150 min of moderate-intensity endurance exercise, two strength sessions, and some flexibility exercise per week [150,180,181]. However, the impact of this type of program and its effect on cognition and brain health has not been evaluated. Ongoing studies hope to determine its impact [243]. It is also unclear how alternative forms of exercise, such as yoga, tai chi, or HITT, play into the public health recommendations and how they influence cognitive function and brain health when combined with more traditional forms of exercise (i.e., endurance or resistance training).

### 4.7. Exercise Intervention Summary

Overall, the literature suggests that exercise and physical activity positively affect cognitive function and brain health. Unfortunately, it is not clear what exercise should be prescribed to maintain and potentially enhance cognition and brain health with age. Continued research in this area strives to answer these questions. Table 2 provides a brief overview of each dietary intervention approach described above.

## 5. Conclusions

Age-related changes in cognition, brain structure, and physiology are potential targets for lifestyle interventions. Improving health through diet and exercise may prevent or delay cognitive decline related to Alzheimer’s and dementia. Dietary patterns are an essential factor in cognitive and brain health for aging individuals [60]. Promising research utilizing various dietary approaches, including the MeDi, DASH, MIND, Ketogenic, intermittent fasting, and weight loss diets, is available. Common targets of all diets addressed include inflammation, oxidation, glucose metabolism, insulin sensitivity, and adiposity, all factors associated with aging and considered risk factors for Alzheimer’s disease and related dementias. In addition to diet, exercise and physical activity also positively affect cognitive function and brain health. Depending on type and intensity, exercise can target brain vascularization, neurotransmitter regulation, growth factors, and neurogenesis [255]. Unfortunately, it is not clear what exercise should be prescribed to maintain and potentially enhance cognition and brain health with age. Also, the interaction between dietary interventions and exercise and their effects on aging, cognition, and other risk factors for Alzheimer’s disease and related dementias (ADRD) has not been established. Additionally, it will be essential to consider the impact non-modifiable risk factors for ADRD have on the effectiveness of these interventions. While this is a common practice for some non-modifiable risk factors such as genetics (e.g., APOE status) [256], others, such as sex, are not as widely examined in this context [257].

## Figures and Tables

**Table 1:** Summary of dietary interventions that support cognition and brain health in older adults.

Dietary Intervention	Key Characteristics/Focus	Associated Cognitive Benefits	Associated Brain Health Benefits
**Mediterranean Diet (** **MeDi)**	Increase intake of vegetables, fruits, nuts, seeds, low-fat dairy	Higher general cognition, decreased risk of developing dementia, decreased risk of converting from MCI to AD; positive association with working memory, verbal fluency, attention, and long-term memory	Cell membrane and vascular integrity, inflammation, resolution, and oxidation; lipid & energy metabolism
**Dietary Approaches to Stop Hypertension (** **DASH)**	Increase intake of vegetables, fruits, and whole grains, nuts, low-fat dairy, and reduction of sodium intake	Higher general cognition	Cell membrane and vascular integrity
**Mediterranean-DASH Intervention for Neurodegenerative Delay** **(MIND)**	Increase intake of leafy greens, berries, fatty fish, and olive oil	Higher general cognition, decreased risk of developing dementia; positive association with working memory, verbal fluency, visuospatial ability, perceptual speed, and executive function	Cell membrane and vascular integrity, inflammation, resolution, and oxidation; lipid & energy metabolism
**Ketogenic Diet**	Switching the body’s energy consumption to ketones instead of glucose by limiting intake of sugar and simple carbohydrates while increasing protein intake	Higher general cognition; positive association with episodic memory	Neurotransmitter regulation, synaptic maintenance, and reduction of oxidation in animal studies
**Intermittent Fasting**	Keeping the body in a fasted state for 12 or more hours per day, aiding in restriction of caloric intake	Higher general cognition	Neurotransmitter regulation, synaptic maintenance, and reduction of oxidation in animal studies
**Weight Management Diets**	Reducing caloric intake by adjusting ratio of carbohydrates and fats or adopting a heart healthy diet	Executive function, memory, learning, psychomotor speed	More research is needed

**Table 2:** Summary of exercise interventions that support cognition and brain health in older adults.

Exercise Intervention	Characteristics/Focus	Associated Cognitive Benefits	Associated Brain Health Benefits
**Endurance**	Prolonged physical exertion with energy requirements supplied primarily by endurance metabolism	Reduced rate of cognitive decline, improvement in global cognition, visual attention, and memory	Vascularization, synaptic plasticity, reduced amyloid burden, reduced risk of cardiovascular disease, attenuate atrophy
**Resistance training**	Use of muscular contraction against resistance	Global cognition executive function, memory, verbal fluency	More research is needed
**Combined exercise**	Usually, an exercise program that combines endurance/aerobic exercise and resistance exercise	More research is needed	Improved insulin resistance, reduced inflammation
**Yoga**	Physical postures, rhythmic breathing, and meditative practice	Memory, executive function, attention, and processing speed	Improved brain structure and function
**Tai Chi**	Form of martial arts that emphasizes gentle movements, physical postures, and controlled breathing	Global cognition, executive function	Increased gray matter volume and neural activity, but more research is needed
**High-Intensity Interval Training (HIIT)**	Alternates between high-intensity bursts of aerobic exercise, with short periods of low-intensity movements for recovery	Cognitive flexibility	Improved neuroplasticity and increased BDNF production

## Data Availability

No new data were created or analyzed in this review. Data sharing is not applicable to this article.

## Abbreviations

ACSM = American College of Sports Medicine, AD = Alzheimer’s Disease, ADRD = Alzheimer’s Disease and related dementias, AMP = Adenosine Monophosphate, ATP = Adenosine Triphosphate, BDNF = Brain-Derived Neurotropic Factor, BMI = Body Mass Index, CDC = Centers for Disease Control, CSF = Cerebrospinal Fluid, DASH = Dietary Approaches to Stop Hypertension, DTI = Diffusion Tensor Imaging, FA = Fractional Anisotropy, HIIT = High-Intensity Interval Training, IF = Intermittent Fasting, IGT = Iowa Gambling Task, KD = Ketogenic Diet, LDL = Low-Density Lipoprotein, MCI = Mild Cognitive Impairment, MeDi = Mediterranean Diet, MIND = Mediterranean-DASH Intervention for Neurodegenerative Delay, MMSE = Mini-Mental State Exam, MRI = Magnetic Resonance Imaging, PET = Positron Emission Tomography, PFC = Prefrontal Cortex, PIB = Pittsburgh Compound B, SMD = Standard Mean Difference, WHO = World Health Organization, WM = White Matter, WMH = White Matter Hyperintensities.
